# RIP kinase inhibition with Necrostatin-1 improves human marginal mass islet graft survival and function for the management of type 1 diabetes

**DOI:** 10.1038/s41419-026-08728-8

**Published:** 2026-04-08

**Authors:** Saloni Aggarwal, Nerea Cuesta-Gomez, Joy Paramor, Sandra Kelly, Kateryna Polishevska, Karen Seeberger, Jessica Worton, Purushothaman Kuppan, Andrew R. Pepper

**Affiliations:** 1 Department of Surgery, University of Alberta, Edmonton, AB Canada; 2 Alberta Diabetes Institute, University of Alberta, Edmonton, AB Canada

**Keywords:** Translational research, Necroptosis, Diabetes

## Abstract

Islet transplantation (ITx) has demonstrated that cellular therapies can improve glycemic control in patients with type 1 diabetes. However, cell death in the acute post-transplant period accounts for the loss of up to 70% of the islets, resulting in the requirement of multiple donors per recipient to achieve normoglycemia. Several studies have targeted apoptosis prevention after ITx; herein, our study explores a novel approach by modulating RIPK1-associated stress pathways using Necrostatin-1 (Nec-1) to enhance human islet survival, engraftment, and function post-transplant. Nec-1 treatment for 24 h prior to transplant significantly reduced the expression of RIPK1 (*p* = 0.0021) and RIPK3 (*p* = 0.0042), resulting in decreased cell death (*p* < 0.0001) and necroptosis (*p* < 0.0001), measured as TUNEL^+^ and pMLKL^+^ cells, respectively, without affecting basal respiration or insulin secretion in human islets. Nec-1 treatment pre-transplant drastically reduced cytokine (*p* = 0.0083), NFκB (*p* = 0.0179), TGFβ (*p* = 0.0015) and TNF family (*p* = 0.0010) signaling pathways at the transcriptional level compared to control. These results correlated with increased diabetes reversal (*p* = 0.0200) and decreased reversal time (*p* = 0.0011) in the Nec-1-treated group compared to untreated controls. The success of Nec-1 treatment in this study showcases that short-term modulation of RIPK1-associated stress pathways promotes early human marginal mass engraftment post-ITx. In the clinical context, improvement of marginal mass islet function could enable single-donor islet infusion and could aid in future ß-cell replacement therapies, making ITx available to a broader population of individuals living with diabetes.

## Introduction

Islet transplantation (ITx) improves glycemic control in type 1 diabetes, yet long-term insulin independence remains low, dropping to 20% at 10 years and 8% at 20 years [1]. Up to 70% of transplanted islets are lost in the acute peri-transplant period [2], driven by inflammation-induced apoptosis and necrosis [3–6].

Anti-apoptotic agents improve viability in marginal-dose transplants, but controlling necrosis remains challenging [7–10]. Necroptosis, a regulated form of necrosis mediated by receptor-interacting protein kinases (RIPK1/3) and mixed lineage kinase domain-like protein (MLKL), offers a potential target for intervention [11]. RIPK1/3 inhibitors have been explored in models of ischemia-reperfusion and systemic inflammation but not in ITx [12, 13].

Necrostatins inhibit necroptosis; necrostatin-1 (Nec-1) blocks RIPK1 autophosphorylation and RIP1–RIP3–MLKL signaling [14, 15]. However, its short in vivo half-life (1–2 h) [16] and off-target inhibition of indoleamine 2,3-dioxygenase (IDO) [15, 17, 18] limit translational potential. Nec-1s is a more stable analog lacking IDO inhibition [15, 19], yet Nec-1’s dual activity may confer enhanced anti-inflammatory potential. Herein, we investigate whether pre-transplant Nec-1 or Nec-1s treatment reduces human islet necrosis and improves engraftment in marginal-dose ITx.

## Materials and methods

All methods were approved by and performed in accordance with the University of Alberta’s Human Research Ethics Board (Pro00092479) and Animal Research Ethics Committee (AUP00003230 and AUP00002977) and complied with the Tri-Council Policy Statement: Ethical Conduct for Research Involving Humans (TCPS 2) and Canadian Council on Animal Care guidelines.

### Cell culture

MIN6 cells (Sigma-Aldrich, St. Louis, Missouri, US) were newly acquired and regularly tested to be mycoplasma-free; no additional STR authentication was performed. MIN6 cells were cultured as previously described [20]; 1.0 × 10^6^ MIN6 cells were co-transfected with siRIPK1 or nonspecific siRNA using Lipofectamine 2000 (Thermo Fisher Scientific, Waltham, Massachusetts, US) in OptiMEM for 24 h.

Mouse islets were isolated as described [21], and human islets were obtained from the Alberta Diabetes Institute’s Islet Core (Table 1). MIN6 cells or human islets were incubated with 100 μM of Nec-1 or Nec-1s (Sigma-Aldrich) or 20 μM of Z-VAD (R&D Systems, Minneapolis, Minnesota, US) for 24 h. Cell death was induced with 400 μM H_2_O_2_ for 2 h. MIN6 viability was assessed with trypan blue (Thermo Fisher Scientific), and human islets with TUNEL staining.

**Table 1:** Donor characteristics of the human islets used in this study.

Human islet batch number	Donor age (years)	Sex	BMI	Purity (%)
H2349	69	F	30.1	40
R389	65	F	24.4	75
R403	35	F	37.2	95
H2373	59	M	25.6	37.5
H2387	43	F	31.1	40
R408	39	M	27.5	80
R410	59	F	26.4	80
R412	42	M	29.9	85
R413	41	M	33.1	90
R419	70	M	31.5	80
R420	55	M	23.5	85
R430	49	M	24.4	90
R505	53	F	33.1	80
R536	37	M	28.2	75

### Western blotting

Performed as described [22]. Primary antibodies used were diluted 1:1000 (RIPK1 [Cell Signaling Technology, Danvers, Massachusetts, US] and β-actin [Thermo Fisher Scientific]). Membranes were developed with the LI-COR Odyssey system.

### Glucose-stimulated insulin secretion assay

A total of 5.0 × 10^5^ MIN6 cells or 500 islet equivalents (IEQs) were equilibrated in CMRL for 15 min, then incubated sequentially in 2.8 mM and 24.4 mM glucose for 1 h each. Dynamic insulin secretion was assessed using 40 islets with PERI5 Perifusion System (BIOREP, Miami Lakes, Florida, US) with a flow rate of 0.1 ml min^−1^ and with 6 min low glucose, 20 min high glucose, 20 min low glucose, and 20 min in KCl. Insulin was measured with ELISA (ALPCO, Salem, New Hampshire, US) and normalized per islet.

### NanoString nCounter gene expression assay

RNA was extracted with the RNeasy Micro Kit (Qiagen, Hilden, Germany). Human Organ Transplant panel and Mouse Immunology panels (NanoString Technologies, Seattle, Washington, US) were used as per manufacturer’s instructions and scanned at 555 FOV using the nCounter Digital Analyzer. Quality control, data normalization, and data analysis were performed using nSolver 4.0 (NanoString Technologies).

### Immunohistochemistry

Human islet and graft staining was performed as previously described [22]. Antibodies and concentrations are listed in Table 2. Slides were visualized and processed using Zeiss Observer Z1 and analyzed using QuPath.

**Table 2:** Antibodies and concentrations used for immunohistochemistry.

Epitope	Dilution	Supplier
Insulin	1:5	Dako (Glostrup, Denmark) (A0564)
Glucagon	1:5000	Sigma-Aldrich (G2654)
UCN3	1:100	Abcam (Cambridge, UK) (ab224545)
Phospho-MLKL	1:100	Thermo Fisher Scientific (PA5-105678)
CC3	1:100	Cell Signaling (Danvers, USA) (9664)
STT	1:1000	Dako (A0566)
MAF-A	1:200	Abcam (ab264418)
PDX1	1:200	R&D Systems (AF2419)
NKX6.1	1:20	DSHB (Iowa, USA) (F55A10)
NKX2.2	1:100	Abcam (ab187375)
Anti-guinea pig	1:200	Thermo Fisher Scientific (A11073)
Anti-mouse	1:200	Thermo Fisher Scientific (A11032)
Anti-rabbit	1:200	Thermo Fisher Scientific (A11012)

### Oxygen consumption rate (OCR) assay

Seventy handpicked human islets were analyzed with XF24 Extracellular Flux Analyzer (Seahorse Bioscience, North Billerica, Massachusetts, US). Basal respiration was measured in 2.8 mM glucose for 30 min, followed by sequential exposure to 16.8 mM glucose, 5 μM Oligomycin A, 3 μM FCCP, and 5 μM Antimycin A/rotenone. Data were standardized to DNA.

### Diabetic induction and transplantation

Diabetes was induced in B6.129S7-Rag1^tm1Mom^(B6/Rag^−/−^) mice (Jackson Laboratory, Bar Harbor, Maine, US) by intraperitoneal injection of 180 mg/kg streptozotocin (Sigma-Aldrich). Mice were between 8 and 12 weeks old and balanced for sex. After 24-h culture with inhibitors, 500 human islets were transplanted under the left kidney capsule [23]. Sham controls received surgery without transplantation. Animals were randomly assigned, and follow-up was performed by a blinded investigator. At 60 days post-transplantation, mice underwent graft nephrectomy and were monitored for hyperglycemia. Only animals reverting to hyperglycemia following graft removal were included. Each group included six mice, except Z-VAD and Nec-1 (five each). Sample size was determined based on pilot data (*α* = 0.05, power = 0.8). Alternatively, animals (*n* = 3/group) underwent nephrectomy at day 7 for NanoString analysis.

### Assessment of glycaemic control

Non-fasting blood glucose was measured twice weekly. Intraperitoneal glucose tolerance tests (IPGTT) were conducted 4 weeks post-transplant; blood samples were collected before and 60 min post-injection for human insulin ELISA (ALPCO).

### Statistics

Data distribution was assessed to confirm parametric test assumptions. Between-group comparisons used the *t*-test or one-way ANOVA; two-way ANOVA compared time courses. Kaplan–Meier survival curves were analyzed with log-rank testing (Mantel–Cox). Continuous data are mean ± SEM; discrete data are represented as counts and percentages. Analysis was completed using GraphPad Prism 9.3.1.

## Results

### Nec-1 treatment improves human islet survival and preserves insulin content during islet culture

Seven days post-transplantation of 500 BALB/c islets into diabetic C57BL/6 mice, *RIPK1* and *RIPK3* were significantly upregulated in the graft (Supplementary Fig. S1A, B). Silencing RIPK1 in MIN6 cells did not impair viability or insulin secretion but protected against H₂O₂-induced damage and enhanced glucose-stimulated insulin secretion (Supplementary Fig. S1C–F), indicating RIPK1 inhibition improves β-cell survival and function in cytotoxic environments.

**Fig. 1:**
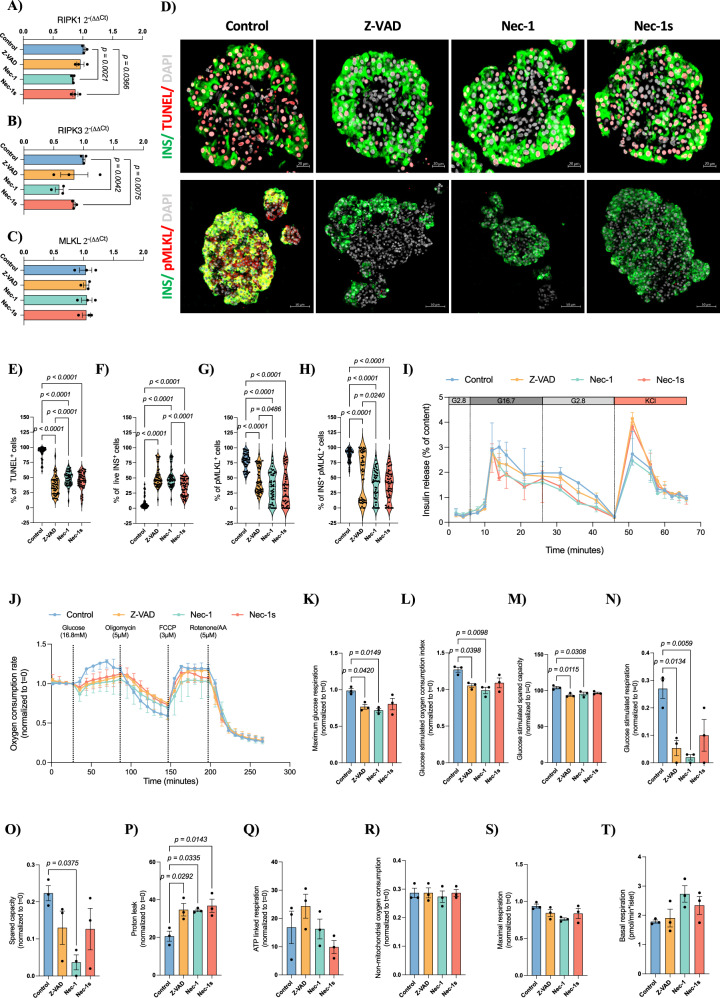
Nec-1 treatment improves islet survival and preserves insulin content during islet culture. **A** Relative *RIPK1*, **B** *RIPK3*, and **C** *MLKL* expression upon human islets cultured with cell death inhibitors for 24 h (*n* = 3 human islet batches per group). **D** Representative immunohistochemistry and quantification of the percentage of **E** TUNEL^+^ cells, **F** live INS^+^ cells, **G** pMLKL^+^ cells, **H** INS^+^ pMLKL^+^ cells of human islets cultured with cell death inhibitors for 24 h (*n* = 50 islets from three human islet batches per group). **I** Insulin release in response to glucose and KCl in human islets cultured with cell death inhibitors for 24 h (*n* = 3 biological replicates per group). **J** Measurement of oxygen consumption rate, **K** maximum glucose respiration, **L** glucose stimulated oxygen consumption index, **M** glucose stimulated spared capacity, **N** glucose stimulated respiration, **O** spared capacity, **P** proton leak, **Q** ATP linked respiration, **R** maximal respiration, **S** non-mitochondrial oxygen consumption normalized to time 0 of upon human islets cultured with cell death inhibitors for 24 h and **T** basal respiration per islet (*n* = 3 biological replicates per group). Between-group comparisons were carried out using a one-way ANOVA test. All data are represented as mean ± SEM.

Human islets were cultured for 24 h with Nec-1 or Nec-1s to assess the effect of pharmacological necroptosis inhibition; the apoptosis inhibitor Z-VAD served as a positive control for cell death inhibition. All experiments were performed on naïve islets, as human islets in culture exhibit progressive cell death under standard conditions [24]. RIPK1 and RIPK3 expression was significantly downregulated in Nec-1 (RIPK1: Control: 1.02 ± 0.02, Nec-1: 0.83 ± 0.01, *p* = 0.0021; RIPK3: Control: 1.00 ± 0.02, Nec-1: 0.59 ± 0.06, *p* = 0.0042) and Nec-1s (RIPK1: Nec-1s: 0.87 ± 0.04, *p* = 0.0366; RIPK3: Nec-1s: 0.84 ± 0.02, *p* = 0.0075) treated islets (Fig. 1A, B); MLKL levels were unchanged (Fig. 1C). All inhibitors reduced TUNEL^+^ cells (Control: 91.10 ± 1.49; Z-VAD: 36.61 ± 2.15, *p* < 0.0001; Nec-1: 48.32 ± 1.61, *p* < 0.0001; Nec-1s:44.35 ± 2.00, *p* < 0.0001; Fig. 1D, E) and increased live INS^+^ cells (Control: 8.63 ± 1.39; Z-VAD: 51.23 ± 2.34, *p* < 0.0001; Nec-1: 48.85 ± 2.09, *p* < 0.0001; Nec-1s: 34.15 ± 1.90, *p* < 0.0001; Fig. 1D, F). Culture with Nec-1 had an improved effect, preserving the viability of INS^+^ cells than Nec-1s (*p* < 0.0001). All cell death inhibitors reduced the number of cells positive for the active form of MLKL, pMLKL (Control: 77.48 ± 1.85; Z-VAD: 46.13 ± 2.93, *p* < 0.0001; Nec-1: 34.68 ± 3.38, *p* < 0.0001; Nec-1s:34.71 ± 3.89, *p* < 0.0001; Fig. 1D, G), and INS^+^ pMLKL^+^ cells (Control: 87.94 ± 1.38; Z-VAD: 53.52 ± 5.23, *p* < 0.0001; Nec-1: 38.96 ± 3.38, *p* < 0.0001; Nec-1s: 39.21 ± 3.33, *p* < 0.0001; Fig. 1D, H). Neither Nec-1 nor Nec-1s altered CC3 expression, whereas Z-VAD reduced CC3^+^ (Control: 90.12 ± 1.04; Z-VAD: 82.93 ± 1.42, *p* < 0.0001; Supplementary Fig. S2A, B) and INS^+^CC3^+^ cells (Control: 95.25 ± 0.71; Z-VAD: 91.69 ± 1.04, *p* = 0.0094; Supplementary Fig. S2A, C). Cell death inhibitors did not alter islet composition, measured as INS^+^, INS^+^UCN3^+^, GCG^+^, and SST^+^ cells (Supplementary Fig. S2A, D–G).

Static (Supplementary Fig. S2H–J) and dynamic (Fig. 1I) tests of insulin secretion confirmed glucose responsiveness in all groups. OCR assessment of human islets upon treatment showed metabolic differences in response to glucose (Fig. 1J). Z-VAD and Nec-1 treatment resulted in decreased maximum glucose respiration (Control: 0.98 ± 0.02; Z-VAD: 0.76 ± 0.03, *p* = 0.0420; Nec-1: 0.71 ± 0.02, *p* = 0.0149; Fig. 1K), glucose stimulated oxygen consumption index (Control: 1.27 ± 0.03; Z-VAD: 1.05 ± 0.02, *p* = 0.0398; Nec-1:0.98 ± 0.04, *p* = 0.0098; Fig. 1L), glucose stimulated spared capacity (Control: 104.2 ± 1.64; Z-VAD: 93.59 ± 1.53, *p* = 0.0115; Nec-1: 95.40 ± 2.37, *p* = 0.0308; Fig. 1M), glucose simulated respiration (Control: 0.27 ± 0.03; Z-VAD: 0.05 ± 0.02, *p* = 0.0134; Nec-1: 0.02 ± 0.01, *p* = 0.0059; Fig. 1N) and spared capacity (Control: 0.22 ± 0.03; Z-VAD: 0.13 ± 0.07; Nec-1: 0.03 ± 0.03, *p* = 0.0375; Fig. 1O) and increased proton leak (Control: 20.6 ± 4.5; Z-VAD: 34.7 ± 5.7, *p* = 0.0292; Nec-1: 34.3 ± 1.1, *p* = 0.0335; Fig. 1P). ATP-linked respiration, non-mitochondrial oxygen consumption, and maximal or basal respiration were unaffected (Fig. 1Q–T).

Overall, Nec-1 and Nec-1s improve islet viability without impairing glucose-stimulated insulin secretion, with Nec-1 showing superior protection.

### Nec-1 inhibition downregulates inflammatory pathways in human islets

To evaluate the mechanism by which Nec-1 and Nec-1s promote islet survival NanoString nCounter Human Organ Transplant panel was used.

**Fig. 2:**
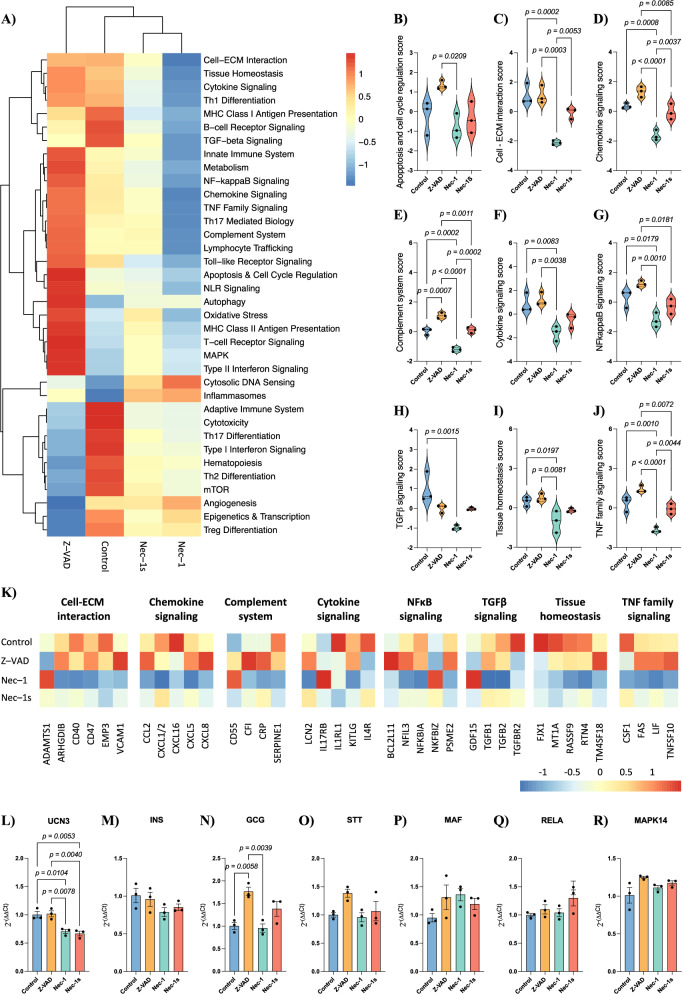
Nec-1 treatment downregulates inflammatory pathways upon culture with human islets. **A** Heatmap representation of transcriptional pathway scores of human islets cultured with cell death inhibitors for 24 h. **B** Quantification of the apoptosis and cell cycle regulation score, **C** cell-extracellular matrix interaction score, **D** chemokine signaling score, **E** complement system score, **F** cytokine signaling score, **G** NFκB signaling score, **H** TGFβ signaling score, **I** tissue homeostasis score and **J** TNF family signaling score of human islets cultured with cell death inhibitors for 24 h (*n* = 3 human islet batches per group). **K** Heatmap representation of key genes differentially expressed upon human islets culture with cell death inhibitors for 24 h. **L** Relative *UCN3*, **M** *INS*, **N** *GCG*, **O** *STT*, **P** *MAF*, **Q** *RELA*, and **R** *MAPK14* expression of human islets cultured with cell death inhibitors for 24 h (*n* = 3 human islet batches per group). Between group comparisons were carried out using one-way ANOVA test. All data are represented as mean ± SEM.

Gene set analysis scores represented as a heatmap showcased differences in signaling pathway transcription (Fig. 2A). Z-VAD, Nec-1, or Nec-1s did not significantly influence the apoptosis or cell cycle regulation pathway scores compared to control; however, Nec-1-treated islets showed a lower pathway score than Z-VAD (*p* = 0.0209; Fig. 2B). Pathway scores reflect transcriptional modulation of genes associated with apoptosis and cell-cycle regulation, rather than the actual extent of apoptosis or proliferation. The higher pathway score observed in Z-VAD–treated islets likely represents increased transcriptional activity of apoptosis-related genes downstream of caspase inhibition, whereas Nec-1, by targeting RIPK1 upstream, results in lower transcriptional changes in these genes. A detailed assessment of genes involved in apoptosis, cell cycle regulation, and proliferation can be found in Supplementary Fig. S3A–C. Nec-1 drastically reduced the significance score of the signaling pathways involved in cell-extracellular matrix (ECM) interaction (*p* = 0.0002; Fig. 2C), chemokine signaling (*p* = 0.0008; Fig. 2D), complement system (*p* = 0.0002; Fig. 2E), cytokine signaling (*p* = 0.0083; Fig. 2F), NFκB (*p* = 0.0179; Fig. 2G), TGFβ (*p* = 0.0015; Fig. 2H), tissue homeostasis (*p* = 0.0197; Fig. 2I) and TNF family (*p* = 0.0010; Fig. 2J). Nec-1s had minor effects on most pathways but increased significance in cell-ECM interaction (*p* = 0.0053; Fig. 2C), chemokine signaling (*p* = 0.0037; Fig. 2D), complement system (*p* = 0.0002; Fig. 2E), and TNF family (*p* = 0.0044; Fig. 2J) pathways compared to Nec-1.

Transcriptional analysis of key genes from these signaling pathways revealed Nec-1 treatment resulted in a significant downregulation of several genes involved in cell-ECM interaction (*ARGHDIB*, *CD40*, *CD47*, and *EMP3*), chemokine signaling (*CCL2*, *CXCL1/2*, *CXCL16*, *CXCL5*, and *CXCL8*), complement signaling (*CFI*, *CRP*, and *SERPINE1*), cytokine signaling (*LCN2*, *IL1R1*, *KITLG*, and *IL4R)*, NFκB signaling (*NFIL3*, *NFKBIA*, and *PSME2*), TGFβ signaling (*TGFB1*, *TGFB2*, and *TGFBR2*), tissue homeostasis (*FJX1*, *MT1A*, *RASSF9*, and *RTN4*) and TNF family signaling (*CSF1*, *FAS*, *LIF*, and *TNFSF10*) pathways while upregulating *ADAMTS1*, *CD55*, *IL17RB*, *NFKBIZ*, and *GDF15* (Fig. 2K). On the contrary, Nec-1s treatment had little effect on the transcriptional regulation of genes involved in the above-mentioned pathways and resulted in a significant downregulation of a few genes, including *EMP3*, *CRP*, *SERPINE1*, *IL1R1*, *TGFB2*, *TGFBR2*, and *FJX1*. Additionally, we assessed the effect of Nec-1 and Nec-1s treatment on the transcription of genes involved with islet cell identity and function. Nec-1 and Nec-1s treatment significantly downregulated the expression of *UCN3* (Control: 1.0 ± 0.05; Nec-1: 0.70 ± 0.03, *p* = 0.0104; Nec-1s: 0.66 ± 0.04, *p* = 0.0053; Fig. 2L) but did not affect the transcription of *INS* (Fig. 2M), *GCG* (Fig. 2N), *STT* (Fig. 2O), *MAF* (Fig. 2P), *RELA* (Fig. 2Q), or *MAPK14* (Fig. 2R).

Overall, Nec-1 induced a greater transcriptional shift than Nec-1s in pathways associated with inflammation, cell survival, and engraftment.

### Nec-1 treatment enables diabetes reversal in marginal mass human islet graft transplants in diabetic mice

To evaluate in vivo efficacy, diabetic Rag mice received 500 human islets pre-treated for 24 h with Z-VAD, Nec-1, or Nec-1s prior to kidney capsule transplantation.

**Fig. 3:**
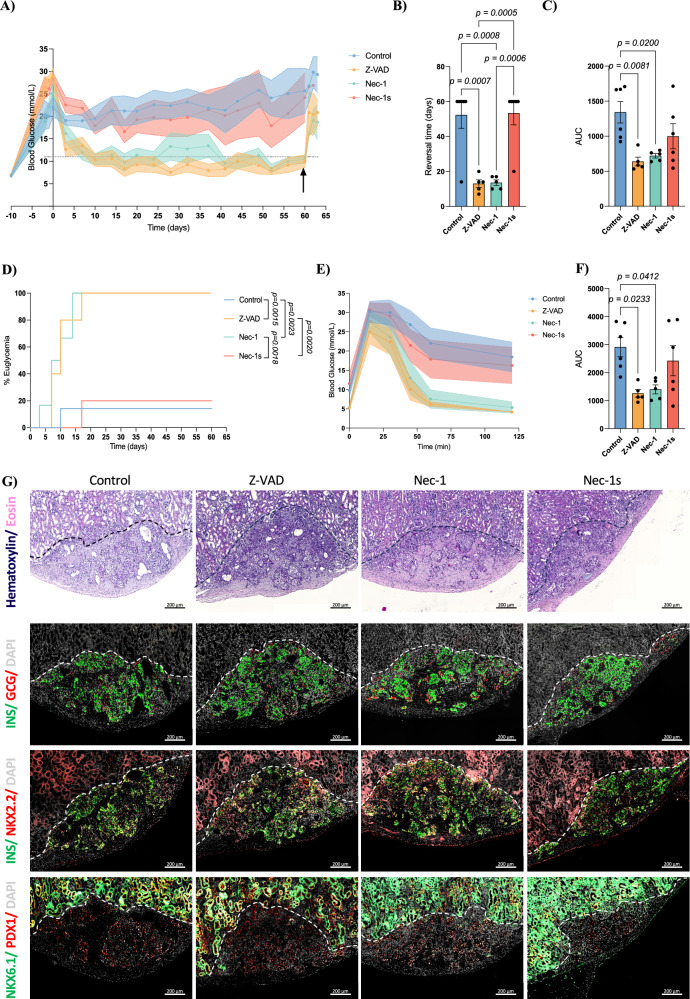
Nec-1 treatment enables diabetes reversal in marginal mass islet graft transplants in diabetic mice. **A** Blood glucose measurements throughout the experiment. Black arrow represents a recovery nephrectomy. **B** Representation of diabetes reversal time. **C** Area under the curve of the blood glucose measurements throughout the experiment. **D** Percentage of euglycemic animals throughout the experiment. **E** Variations in glucose levels during IPGTT at 4-weeks post-transplant. **F** Area under curve for IPGTT at 4-weeks post-transplant. **G** Representative histological images of the collected grafts. In all data 6 animals per group were used for control and Nec-1s groups and 5 animals per group in Z-VAD and Nec-1 groups. Between group comparisons were carried out using one-way ANOVA test. Two-way ANOVA was used to compare time courses. Kaplan–Meier survival curves were compared via log-rank statistical testing (Mantel–Cox). All data are represented as mean ± SEM.

Non-fasting blood glucose levels of the transplanted mice were monitored twice a week to assess diabetes reversal (Fig. 3A). Mice transplanted with control human islets remained hyperglycemic (>20 mM) for the duration of the experiment while Z-VAD and Nec-1 treated islets reversed hyperglycemia within 14 days (Control: 52.33 ± 7.66 days; Z-VAD: 13 ± 2.19 days, *p* = 0.0007; Nec-1: 13.60 ± 1.56 days, *p* = 0.0008; Fig. 3B). Nec-1s treated islets failed to normalize glucose (>15 mM). Recovery nephrectomies at day 60 showed graft-dependent function. Area under the curve (AUC) analysis showed improved glucose control with Z-VAD and Nec-1 (Control: 1341 ± 153.4; Z-VAD: 639.1 ± 61.29, *p* = 0.0081; Nec-1: 719.9 ± 31.67, *p* = 0.0200; Fig. 3C). A total of 100% of the mice transplanted with Z-VAD or Nec-1 treated islets achieved normoglycemia compared to 16.6% of the mice receiving control (Z-VAD: *p* = 0.0015; Nec-1: *p* = 0.0023) or Nec-1s treated islets (Z-VAD: *p* = 0.0020; Nec-1: *p* = 0.0018; Fig. 3D). IPGTT performed at 4 weeks post-transplantation revealed that Nec-1 and Z-VAD treatment improved glucose clearance compared to control (Fig. 3E), as confirmed by their AUCs (Control: 2910 ± 341.9; Z-VAD: 1259 ± 137.9, *p* = 0.0233; Nec-1: 1402 ± 161.1, *p* = 0.0412; Fig. 3F). Representative images of hematoxylin and eosin, insulin and glucagon, insulin and NKX2.2 and PDX1 and NKX6.1 staining of the collected grafts are shown in Fig. 3G; quantification of these markers can be found in Supplementary Fig. S4A–E.

To evaluate whether the protective effects of Z-VAD and Nec-1 are additive, we transplanted human islets pre-treated with either agent alone or in combination under the kidney capsule of diabetic mice (Supplementary Fig. S5A–F). The combined treatment did not confer additional benefit over single-agent pre-treatment, indicating that Z-VAD and Nec-1 do not act synergistically to enhance islet survival in this model.

In summary, diabetes reversal is achievable following pre-treatment of human islets with Z-VAD and Nec-1, but not Nec-1s.

### Nec-1 promotes acute tissue homeostasis, enabling long-term diabetes reversal in marginal mass diabetic mice

To elucidate mechanisms underlying improved engraftment, human islet grafts were collected 7 days post-transplant, homogenized, and analyzed using the NanoString nCounter Human Organ Transplant panel.

**Fig. 4:**
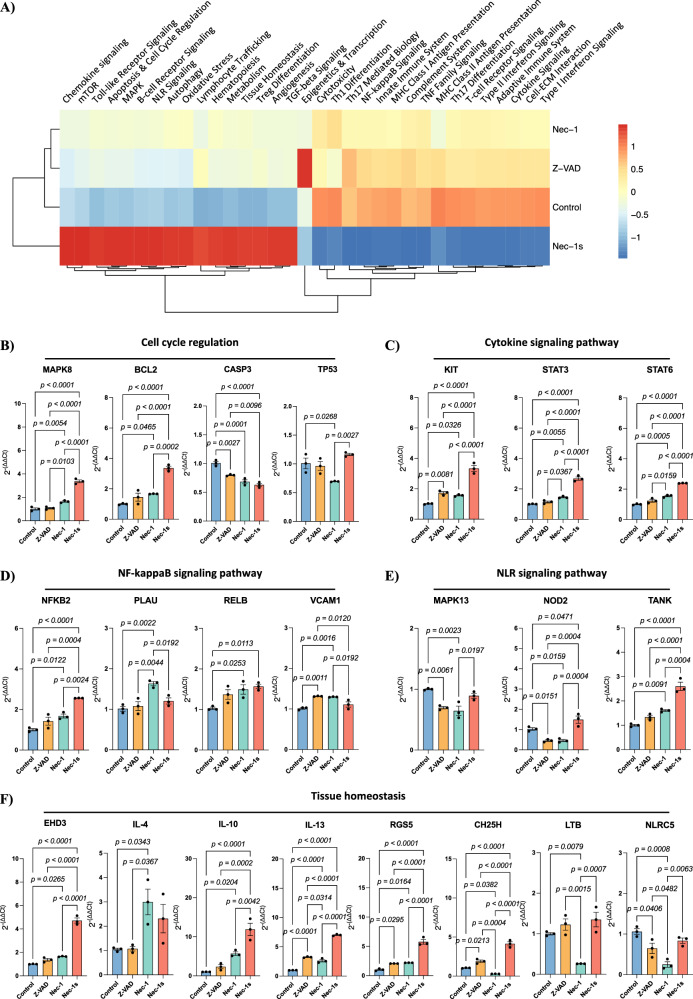
Nec-1 treatment promotes acute tissue homeostasis, enabling long-term diabetes reversal in marginal mass diabetic mice. **A** Heatmap representation of transcriptional pathway scores of human cells within grafts 7 days post-transplant of human islets cultured with cell death inhibitors for 24 h. **B** Relative quantification of key genes involved in cell cycle regulation, **C** cytokine signaling, **D** NFκB signaling, **E** NLR signaling, and **F** tissue homeostasis of human cells within grafts 7 days post-transplant of human islets cultured with cell death inhibitors for 24 h (*n* = 3 animals per group). Between group comparisons were carried out using one-way ANOVA test. All data are represented as mean ± SEM.

Nec-1 treatment drastically impacted the transcription of key signaling pathways involved in cell survival and inflammation (Fig. 4A and Supplementary Fig. S6). Nec-1 treatment resulted in a significant upregulation of genes involved in cell survival regulation including *MAPK8* (*p* = 0.0054) and *BCL2* (*p* = 0.0465) while inducing the downregulation of *CASP3* (*p* = 0.0001) and *TP53* (*p* = 0.0268) compared to control (Fig. 4B). Similarly, Nec-1 treatment prior to transplant resulted in the upregulation of the key cytokine signaling pathway genes *KIT* (*p* = 0.0326), *STAT3* (*p* = 0.0055) and *STAT6* (*p* = 0.0005) compared to control (Fig. 4C) as well as *NFKB2* (*p* = 0.0122), *PLAU* (*p* = 0.0022), *RELB* (*p* = 0.0253) and *VCAM1* (*p* = 0.0016), key genes involved in the NFκB signaling pathway (Fig. 4D). Within the NLR signaling pathway, Nec-1 treatment resulted in a significant downregulation of *MAPK13* (*p* = 0.0023) and *NOD2* (*p* = 0.0159), and a significant upregulation of *TANK* (*p* = 0.0091; Fig. 4E). Finally, Nec-1 treatment of human islets prior to transplant had a significant effect in genes involved in tissue homeostasis; *EHD3* (*p* = 0.0265), *IL-4* (*p* = 0.0343), *IL-10* (*p* = 0.0204), *IL-13* (*p* < 0.0001) and *RGS5* (*p* = 0.0164) were significantly upregulated while *CH25H* (*p* = 0.0382), *LTB* (*p* = 0.0079) and *NLRC5* (*p* = 0.0008) were significantly downregulated upon treatment with Nec-1 (Fig. 4F).

Overall, human islet treatment with Nec-1 for 24 h prior to transplant had a remarkable impact in promoting islet survival and function, as demonstrated by the profound changes in transcriptomics in key pathways involved in graft survival at 7 days post-transplant.

## Discussion

While both apoptosis and necrosis induce β-cell death and graft dysfunction, the unregulated nature of necrosis limits its therapeutic potential. Herein, we investigated whether short-term ex vivo modulation of RIPK1-associated pathways using Nec-1 or Nec-1s could reduce islet death, enhance engraftment, and serve as a targeted therapeutic strategy augmenting β-cell replacement therapies.

Increased RIPK1 and RIPK3 expression following ITx in a mouse allograft model indicated that the necroptotic machinery was active. siRNA-mediated RIPK1 knockdown in MIN6 cells improved survival and function under stress, indicating involvement in β-cell death. Because pharmacological inhibition is more practical than siRNA (faster uptake, lower cost), we pre-treated human islets with Nec-1 and Nec-1s for 24 h before transplantation; recipient mice were untreated. Both inhibitors reduced cell death and necroptosis, as measured by TUNEL⁺ and pMLKL⁺ cells. However, only Nec-1 pre-treatment reversed diabetes in a marginal-mass ITx model.

Immunohistochemistry of recovered grafts showed that Z-VAD and Nec-1 increased INS⁺, GCG⁺, PDX1⁺, PDX1⁺NKX6.1⁺, and INS⁺NKX2.2⁺ cells, indicating preserved endocrine identity and β-cell maturity. Although RIPK1 inhibition reduced *UCN3* expression, a marker of mature β-cells, protein levels remained unchanged, suggesting enhanced insulin secretion did not result solely from altered glucose sensitivity [25]. Interestingly, while Z-VAD is a pan-caspase inhibitor, we observed reduced pMLKL in Z-VAD-treated islets. This finding likely reflects indirect effects of caspase inhibition on upstream cellular stress rather than direct suppression of necroptosis, potentially through reduced ROS, indirectly limiting MLKL phosphorylation [26, 27]. CC3 quantification showed that Z-VAD, but not Nec-1 or Nec-1s, reduced CC3 levels, confirming caspase-inhibition, while high CC3 in cultured islets reflects stress-associated caspase activation rather than true apoptosis execution [28–30].

Because human β-cells express very low RIPK3 and MLKL, their intrinsic capacity to undergo canonical necroptosis is likely limited [31–33]. Accordingly, pMLKL staining alone cannot be considered definitive evidence of necroptosis in human islets. A limitation of this study is that we did not include rigorous biochemical or genetic controls to validate the specificity of pMLKL immunostaining in our experimental system, such as stimulation with established necroptotic inducers (e.g., TNFα/Smac mimetic/zVAD) [34–36], or MLKL silencing strategies [37, 38]. Therefore, reduced pMLKL staining observed following Nec-1, Nec-1s, or Z-VAD treatment may reflect altered RIPK1-dependent stress signaling, changes in cell viability, or nonspecific antibody binding in damaged cells, rather than selective inhibition of necroptosis.

Transcriptional profiling revealed that Z-VAD modulated numerous apoptosis- and stress-related genes, yielding higher apoptosis pathway scores than control or Nec-1-treated islets. Z-VAD inhibits caspase enzymatic activity post-translationally, but upstream stress persists, triggering compensatory transcription. In contrast, Nec-1 acts upstream on RIPK1-dependent stress and cell-death signaling, preventing the initiation of these stress responses and resulting in lower apoptosis pathway scores. These data highlight the importance of distinguishing transcriptional stress signatures from apoptosis execution. Taken together, our findings support a role for RIPK1 as a key mediator of islet stress and death but underscore the need for caution when interpreting pMLKL as a necroptosis-specific marker in human β-cells.

To explain the differential effects of Nec-1 and Nec-1s in vitro and in vivo, we examined downstream RIPK1/3 signaling and cell death pathways triggered by islet isolation, culture, and transplantation. Although the kidney capsule avoids immediate blood-mediated inflammatory reaction (IBMIR), cytokine release and oxidative stress from isolation and culture, and surgical injury activate cell death pathways [39, 40]. RIPK1, typically activated via TNF signaling, also initiates NF-κB/IKK signaling, balancing inflammation and survival [41]. Depending on context, NF-κB may support or oppose RIPK1 effects. Activation promotes genes like BCL2L11 and NFIL3, driving inflammasome activation and death. Conversely, RIPK1 inhibition upregulates NFκBIZ, suppressing NF-κB and promoting survival [15]. This correlates with decreased expression of complement, cytokine, and chemokine genes, and pathways linked to leukocyte infiltration (e.g., NFκB, TGFβ, TNF), ECM remodeling, and homeostasis following 24-h Nec-1/1s treatment. Nec-1/1s also induced anti-inflammatory genes, including ADAMTS1 (remodels ECM, inhibits angiogenesis, and supports proliferation) and CD55 (protects against complement-mediated lysis). Despite similar reductions in β-cell death and improved in vivo function, Nec-1 and Z-VAD transcriptional signatures diverged significantly, reflecting complex, dual roles in inflammation, immune regulation, and cell cycling. Both drastically downregulated mTOR signaling, initially beneficial for glucose metabolism but ultimately associated with dysfunction and apoptosis [42], consistent with better insulin secretion and OCR in control islets in vitro, but poorer in vivo function. These transcriptional changes likely reflect Nec-1-mediated RIPK1-driven inflammatory pathway modulation rather than necroptosis inhibition.

Both Nec-1 and Nec-1s effectively block RIP1-RIP3-MLKL signaling, but Nec-1 uniquely inhibits IDO [43], enhancing anti-inflammatory and pro-survival effects [44, 45], and reduces RIP3 expression and phosphorylation, thereby inhibiting downstream RIP3 activity [16, 46, 47]. Nec-1 also prevents ferroptosis independently of RIPK1/IDO [48]. Upregulation of survival-related genes (*MAPK8, BCL2, KIT, STAT3/6, NFκB2, PLAU, RELB, VCAM1*) [49–57] and downregulation of pro-apoptotic genes (*CASP3, TP53, MAPK13, NOD2*) [58–61], along with upregulation of immunomodulatory factors (*TANK, RGS5, IL-4/10/13*) and downregulation of inflammatory mediators (*CH25H, LTB, NLRC5*), indicate a robust pro-survival, anti-inflammatory profile in Nec-1-treated grafts [62–68]. These combined effects likely contribute to Nec-1’s superior performance vs. Nec-1s. At graft collection, Nec-1 and Z-VAD groups exhibited lower blood glucose levels, suggesting transcriptomic differences may partly reflect metabolic status at harvest.

Nec-1 exposure was limited to the 24-h culture period. Because IDO activity during culture can promote β-cell stress via tryptophan depletion and kynurenine accumulation, transient inhibition likely reduced early inflammatory and metabolic stress [69]. In the transplant environment, IDO activity remains intact, preserving its protective immunomodulatory role [70–72], highlighting its context-dependent dual effect: detrimental during culture but beneficial post-transplant. Because our model used immunodeficient recipients, the impact of Nec-1-treated islets in immunocompetent or allogeneic settings remains to be determined. Future studies using PSC-derived islets with targeted disruption of RIPK1, RIPK3, or MLKL will be essential for delineating necroptosis-specific contributions and refining the translational relevance of these pathways in human islet transplantation.

The subcapsular kidney site was chosen for its reproducibility, technical simplicity, and suitability for graft retrieval. Although the intrahepatic portal vein is the clinical standard, the relative size of rodent islets and the small caliber of the rodent portal vein increase risks such as embolization and hepatic ischemia [73, 74]. Consequently, the kidney capsule is widely accepted for rodent ITx studies but does not replicate hepatic exposure to immunosuppressive drugs or the metabolic microenvironment [75]. Future studies are required to determine whether Nec-1 enhances islet survival in the clinically relevant intraportal setting.

Overall, this study demonstrates that 24 h of islet pre-treatment significantly improves post-transplant glycemic control without systemic exposure. While apoptosis inhibition via pre-treatment or systemic delivery has been previously explored [7–10], this is the first study to validate pre-treatment using RIPK1 modulators. Our data show that Nec-1 and Z-VAD provide comparable cytoprotective effects, indicating that Nec-1 represents a viable alternative to Z-VAD for short-term pre-transplant conditioning. Improved early survival, enhanced engraftment, and accelerated diabetes reversal demonstrate that modulating islet-intrinsic stress and inflammatory pathways is sufficient to improve transplant outcomes, consistent with clinical data showing that short-term TNF-α/IL-1 blockade enhances long-term graft function [76]. Nec-1 treatment shows strong potential for enhancing early engraftment in marginal mass ITx, supporting the use of single-donor transplants and enabling the use of low-yield, high-quality islet preparations. This could reduce the need for multiple donors or procedures, decreasing surgical risks and sensitization, and improving the quality of life for transplant recipients.

## Supplementary information

Supplementary

Western blot

## Data Availability

All data reported in this paper will be shared by the lead contact upon request. This paper does not report original code. Any additional information required to reanalyze the data reported in this paper is available from the lead contact upon reasonable request. Please contact apepper@ualberta.ca.
