# Supplementary material for: RIP kinase inhibition with Necrostatin-1 improves human marginal mass islet graft survival and function for the management of type 1 diabetes
Source: Cell Death Dis. 2026 Apr 8;17(1):501. doi: 10.1038/s41419-026-08728-8 (PMC13187314; doi:10.1038/s41419-026-08728-8)
Supplement: Supplementary file 1 — Supplementary [file 41419_2026_8728_MOESM1_ESM.pdf]

Supporting information for

RIP kinase inhibition with Necrostatin-1 improves human marginal mass islet graft survival and function for the management of type 1 diabetes

Saloni Aggarwal *et al.*

*Corresponding author. Email: apepper@ualberta.ca

This PDF file includes:

Figs. S1 to S6

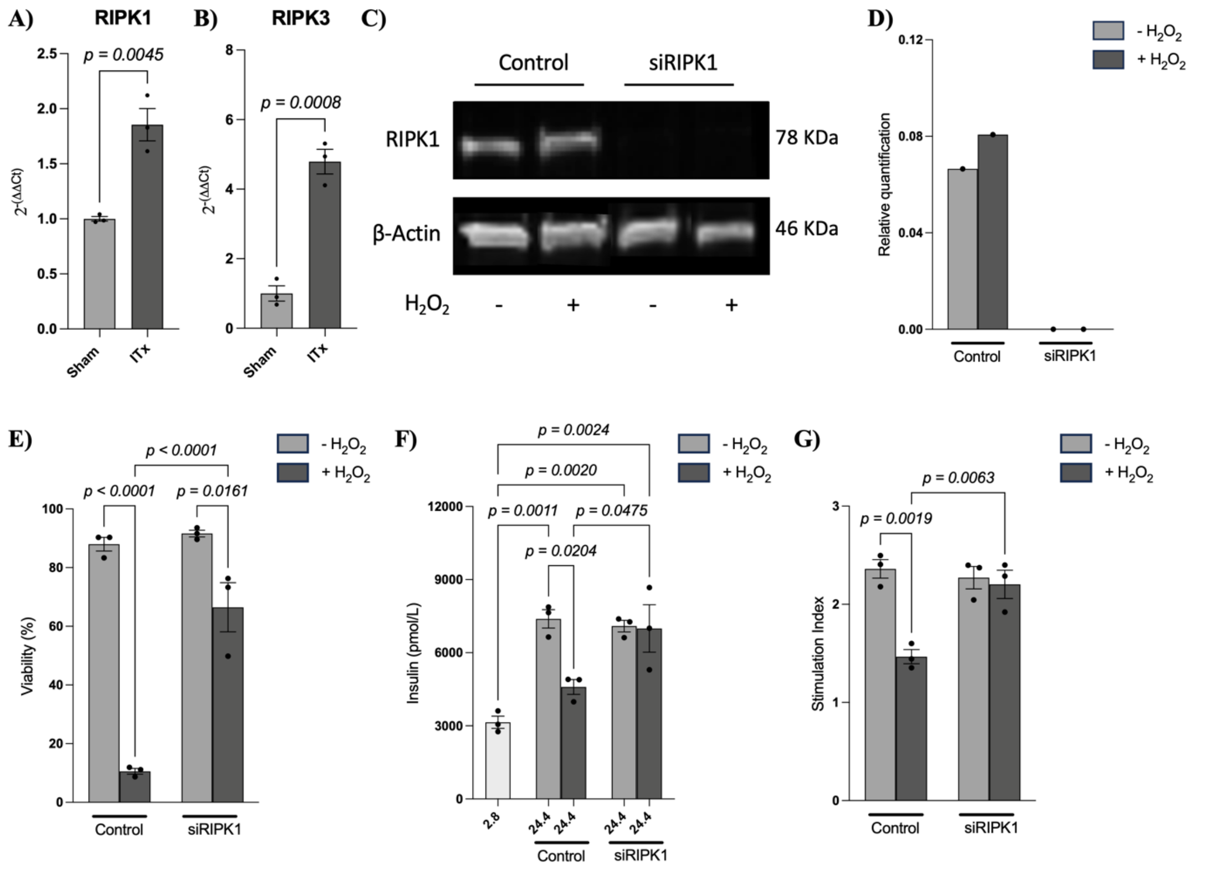

Figure S1. RIPK1 knockdown improves MIN6 cell survival without haltering glucose responsiveness. A) Relative *RIPK1* and B) *RIPK3* expression following allogenic transplant of 500 BALB/c islets delivered into diabetic C57BL/6 mice under the kidney capsule (n=3 animals/ group). C) Representative western blot analyzing RIPK1 protein content in control and siRIPK1 treated MIN6 cells. D) Quantification of the relative density of RIPK1 in control and siRIPK1 treated MIN6 cells. E) Quantification of the percentage of viable cells upon treatment with H_2O_2 in control and siRIPK1 treated MIN6 cells (n=3 biological replicates/ group). F) Quantification of released insulin in the presence of 24.4 mM glucose and g) stimulation index upon treatment with H_2O_2 in control and siRIPK1 treated MIN6 cells. Experiments in C-D were independently repeated three times with similar results. Data in A-B and E-G represent 3 biological experiments. Between

group comparisons were carried out using unpaired t-test. All data are represented as mean with SEM.

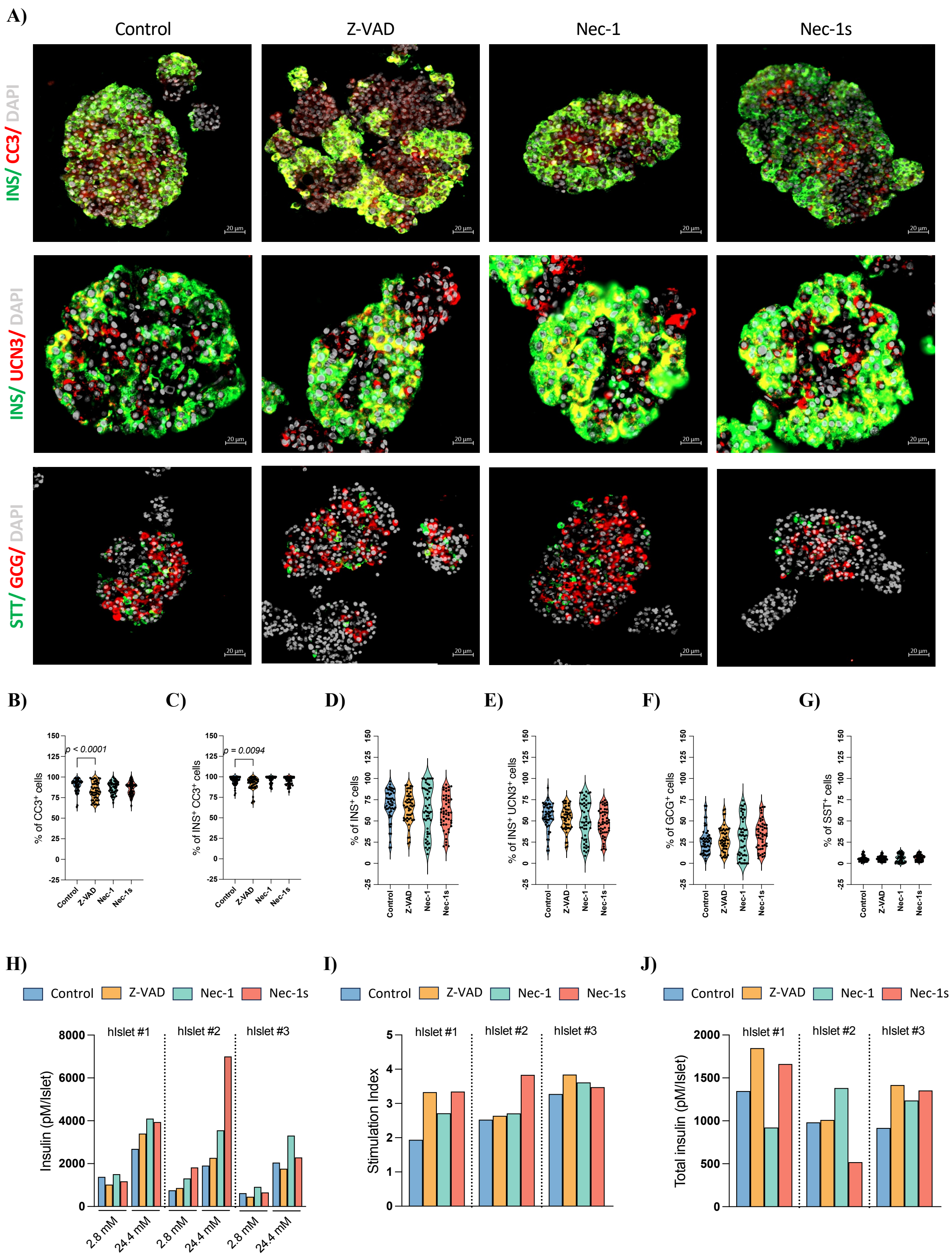

A)
Control
Z-VAD
Nec-1
Nec-1s
INS/ CC3/ DAPI
INS/ UCN3/ DAPI
STT/ GCG/ DAPI
B)
C)
D)
E)
F)
G)
p < 0.0001
p = 0.0094
% of CC3+ cells
% of INS+ CC3+ cells
% of INS+ cells
% of INS+ UCN3+ cells
% of GCG+ cells
% of SST+ cells
H)
I)
J)
Control Z-VAD Nec-1 Nec-1s
hIslet #1
hIslet #2
hIslet #3
Insulin (pM/Islet)
2.8 mM
24.4 mM
Stimulation Index
Total insulin (pM/Islet)

Figure S2. Necroptosis inhibition improves islet survival and preserves insulin content during islet culture. A) Representative immunohistochemistry and quantification of the percentage of B) $CC3^+$ cells, C) INS^+ $CC3^+$ cells, D) INS^+ cells, E) INS^+ $UCN3^+$ cells, F) GCG^+ cells and G) STT^+ cells of human islets cultured with cell death inhibitors for 24 hours (n=50 islets from three human islet batches per group). H) Quantification of released insulin in the presence of 2.8- and 24.4-mM glucose, I) stimulation index and J) total insulin of three independent human islet batches cultured with cell death inhibitors for 24 hours. Between group comparisons were carried out using one-way ANOVA. All data are represented as mean with SEM.

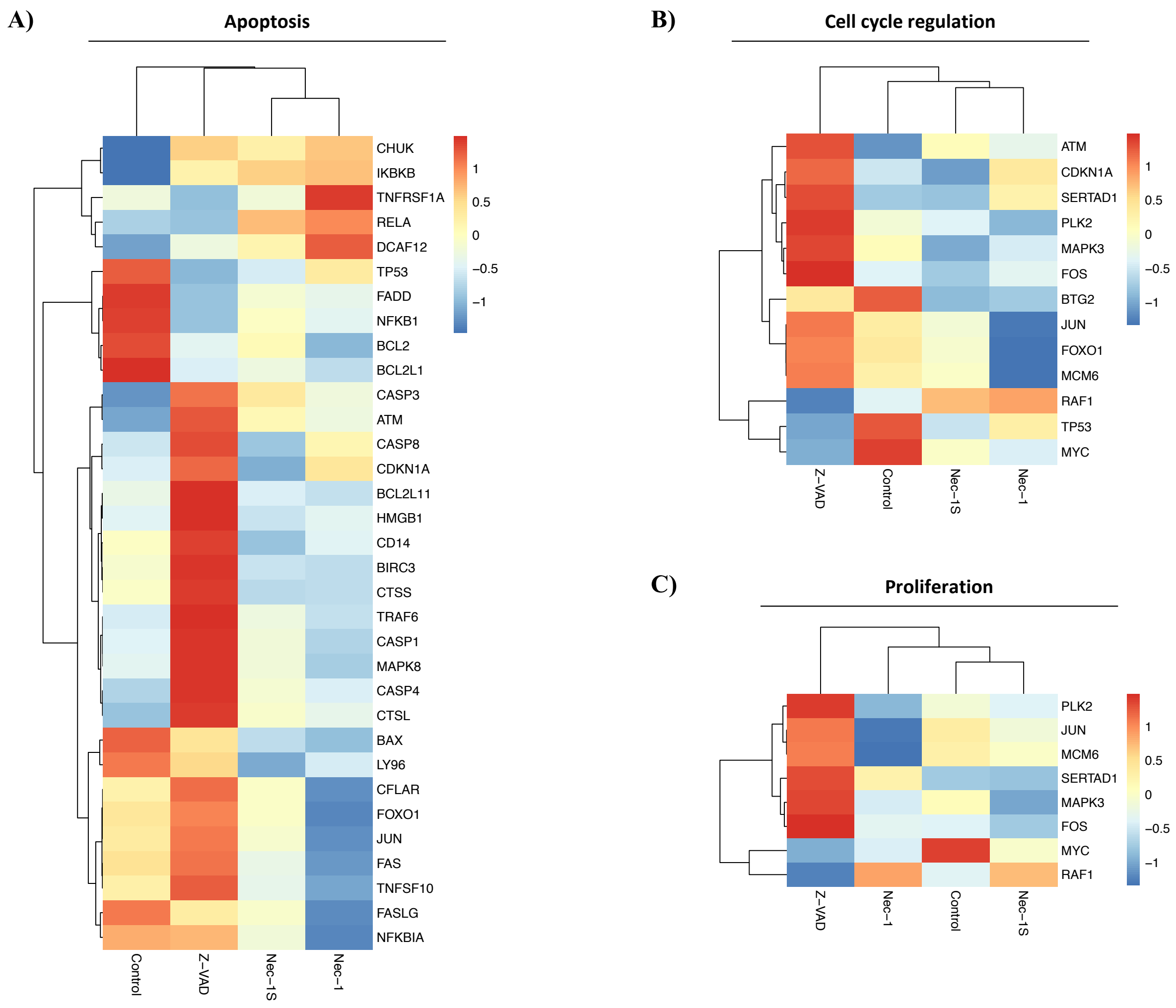

Figure S3. Nec-1 treatment downregulates inflammatory pathways upon culture with human islets. A) Heatmap representation of key genes related to apoptosis, B) cell cycle regulation and C) proliferation that are differentially expressed in human islets cultured for 24 hours with cell death inhibitors.

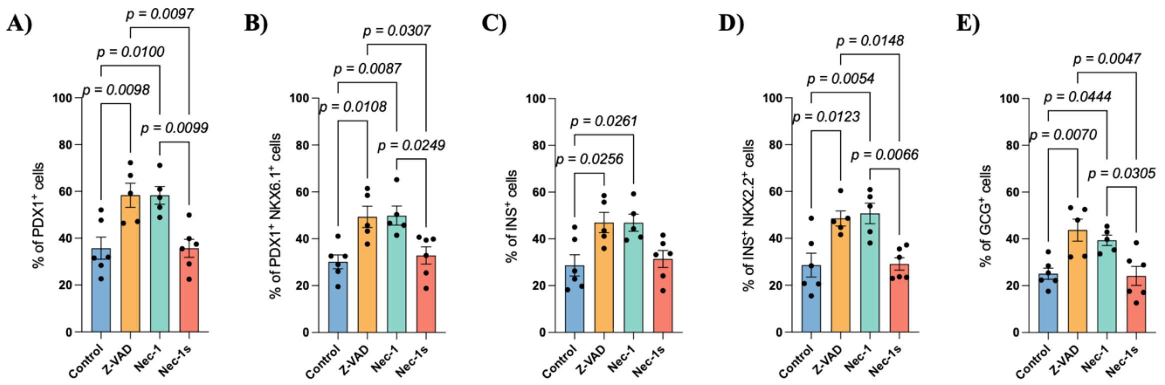

Figure S4. Nec-1 treatment increases endocrine content in the graft. A) Quantification of the percentage of $PDX1^+$ cells, B) $PDX1^+$ $NKX6.1^+$ cells, C) INS^+ cells, D) INS^+ $NKX2.2^+$ cells and E) GCG^+ cells in the grafts collected from transplanted animals. Between group comparisons were carried out using one-way ANOVA. All data are represented as mean±SEM.

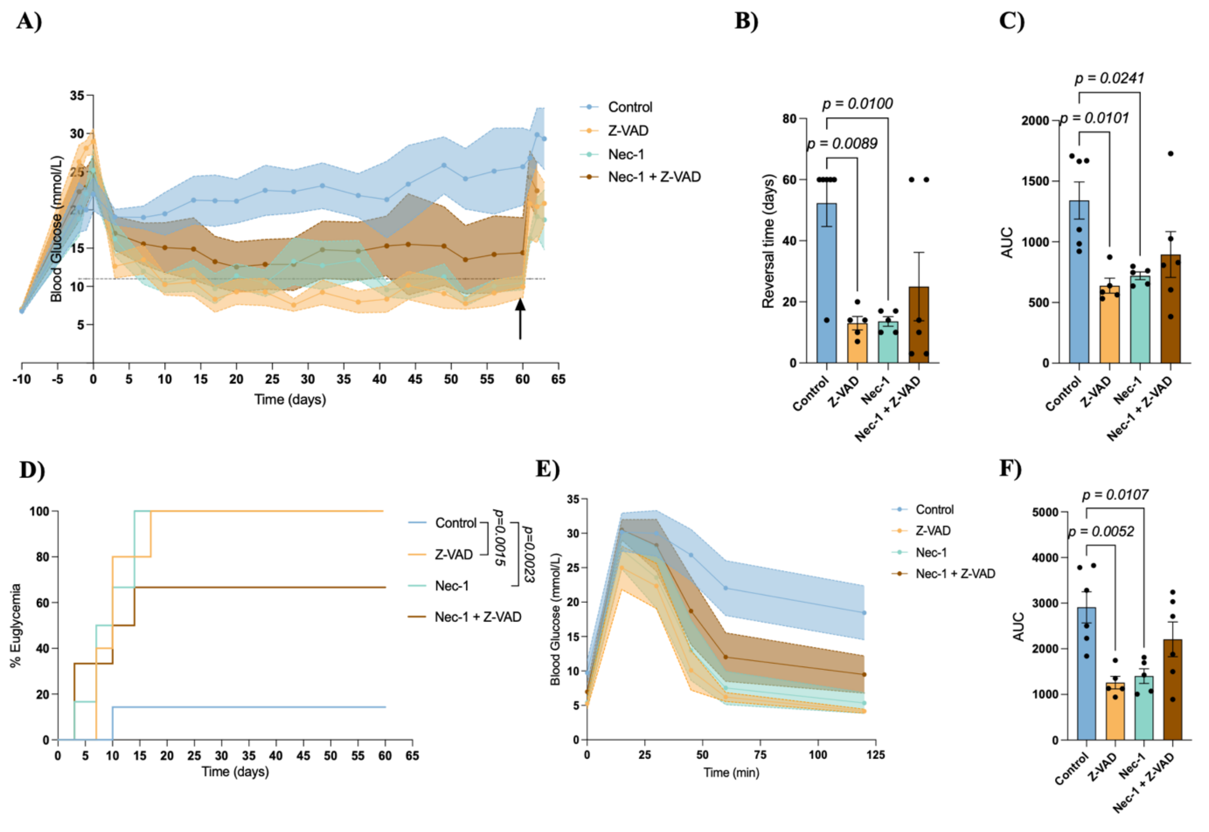

Figure S5. Combined treatment with Nec-1 and Z-VAD does not confer additional benefit. A) Blood glucose measurements throughout the experiment. Black arrow represents recovery nephrectomy. B) Representation of diabetes reversal time. C) Area under the curve of the blood glucose measurements throughout the experiment. D) Percentage of euglycemic animals throughout the experiment. E) Variations in glucose levels during IPGTT at 4-weeks post-transplant. F) Area under curve for IPGTT at 4-weeks post-transplant. Between group comparisons were carried out using one-way ANOVA test. Two-way ANOVA was used to compare time courses. Kaplan-Meier survival curves were compared via log-rank statistical testing (Mantel-Cox). All data are represented as mean±SEM.

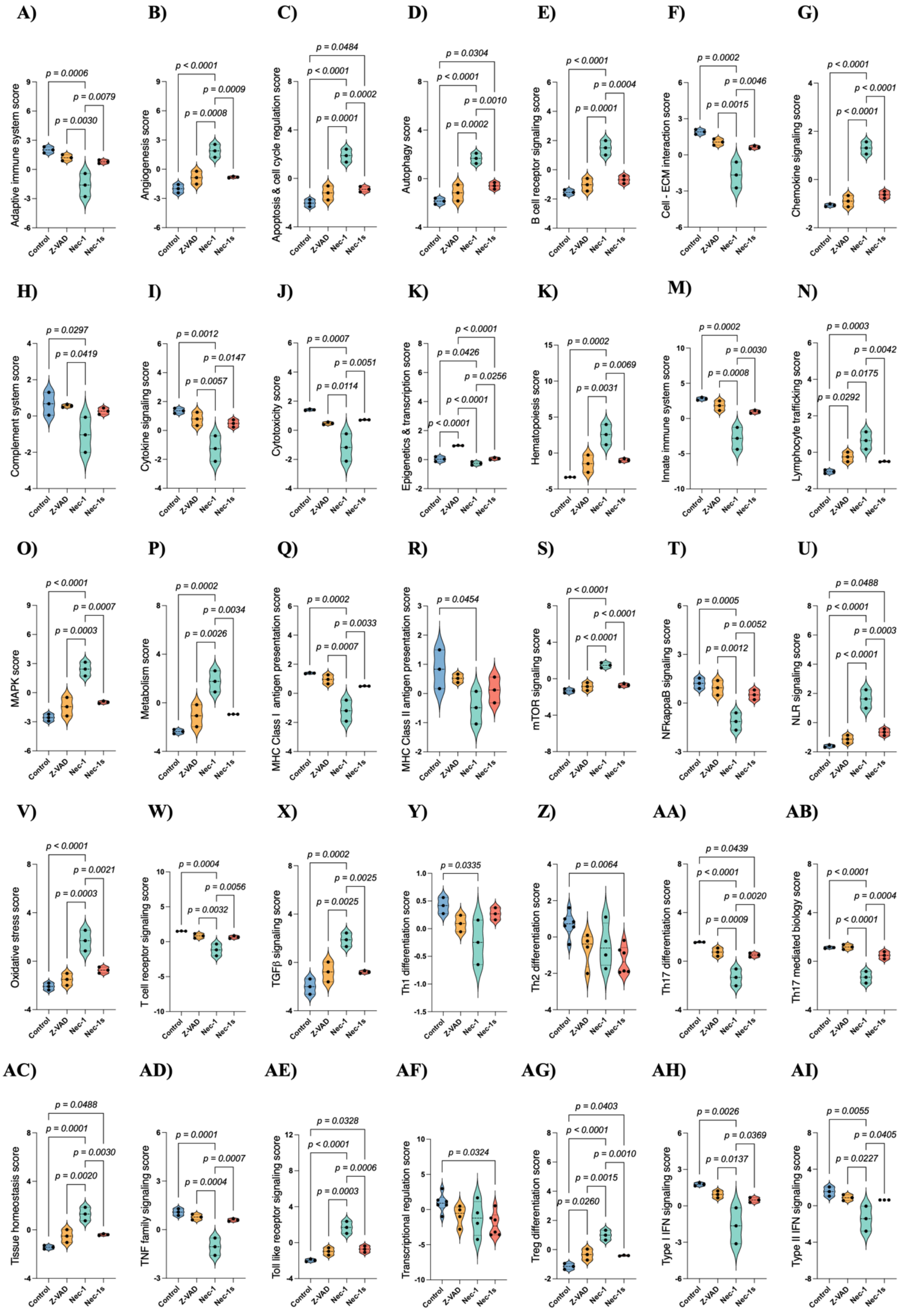

A) Adaptive immune system score
p = 0.0006
p = 0.0079
p = 0.0030
B) Angiogenesis score
p < 0.0001
p = 0.0009
p = 0.0008
C) Apoptosis & cell cycle regulation score
p = 0.0484
p < 0.0001
p = 0.0002
p = 0.0001
D) Autophagy score
p = 0.0304
p < 0.0001
p = 0.0010
p = 0.0002
E) B cell receptor signaling score
p < 0.0001
p = 0.0004
p = 0.0001
F) Cell - ECM interaction score
p = 0.0002
p = 0.0046
p = 0.0015
G) Chemokine signaling score
p < 0.0001
p < 0.0001
p < 0.0001
H) Complement system score
p = 0.0297
p = 0.0419
I) Cytokine signaling score
p = 0.0012
p = 0.0147
p = 0.0057
J) Cytotoxicity score
p = 0.0007
p = 0.0051
p = 0.0114
K) Epigenetics & transcription score
p < 0.0001
p = 0.0426
p = 0.0256
p < 0.0001
p < 0.0001
K) Hematopoiesis score
p = 0.0002
p = 0.0069
p = 0.0031
M) Innate immune system score
p = 0.0002
p = 0.0030
p = 0.0008
N) Lymphocyte trafficking score
p = 0.0003
p = 0.0042
p = 0.0175
p = 0.0292
O) MAPK score
p < 0.0001
p = 0.0007
p = 0.0003
P) Metabolism score
p = 0.0002
p = 0.0034
p = 0.0026
Q) MHC Class I antigen presentation score
p = 0.0002
p = 0.0033
p = 0.0007
R) MHC Class II antigen presentation score
p = 0.0454
S) mTOR signaling score
p < 0.0001
p < 0.0001
p < 0.0001
T) NFkappaB signaling score
p = 0.0005
p = 0.0052
p = 0.0012
U) NLR signaling score
p = 0.0488
p < 0.0001
p = 0.0003
p < 0.0001
V) Oxidative stress score
p < 0.0001
p = 0.0021
p = 0.0003
W) T cell receptor signaling score
p = 0.0004
p = 0.0056
p = 0.0032
X) TGFβ signaling score
p = 0.0002
p = 0.0025
p = 0.0025
Y) Th1 differentiation score
p = 0.0335
Z) Th2 differentiation score
p = 0.0064
AA) Th17 differentiation score
p = 0.0439
p < 0.0001
p = 0.0020
p = 0.0009
AB) Th17 mediated biology score
p < 0.0001
p = 0.0004
p < 0.0001
AC) Tissue homeostasis score
p = 0.0488
p = 0.0001
p = 0.0030
p = 0.0020
AD) TNF family signaling score
p = 0.0001
p = 0.0007
p = 0.0004
AE) Toll like receptor signaling score
p = 0.0328
p < 0.0001
p = 0.0006
p = 0.0003
AF) Transcriptional regulation score
p = 0.0324
AG) Treg differentiation score
p = 0.0403
p < 0.0001
p = 0.0010
p = 0.0015
p = 0.0260
AH) Type I IFN signaling score
p = 0.0026
p = 0.0369
p = 0.0137
AI) Type II IFN signaling score
p = 0.0055
p = 0.0405
p = 0.0227
Control
Z-VAD
Nec-1
Nec-1s

Figure S6. Nec-1 promotes acute tissue homeostasis enabling long term diabetes reversal in marginal mass diabetic mice. A-AI) Quantification of the pathway scores obtained using the NanoString nCounter Human Organ Transplant panel to assess the expression of 36 signalling pathways ranging from inflammation and immune cell regulation to cell cycle regulation and metabolism (n=3 animals per group). Between group comparisons were carried out using one-way ANOVA. All data are represented as mean±SEM.
