# Supplementary figures and images for: RIP kinase inhibition with Necrostatin-1 improves human marginal mass islet graft survival and function for the management of type 1 diabetes
Source: Cell Death Dis. 2026 Apr 8;17(1):501. doi: 10.1038/s41419-026-08728-8 (PMC13187314; doi:10.1038/s41419-026-08728-8)

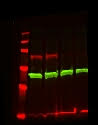

Supplement: Supplementary file 2 — Western blot [file 41419_2026_8728_MOESM2_ESM.jpg]
